# Somatization in Post-Concussion Syndrome: A Retrospective Study

**DOI:** 10.7759/cureus.743

**Published:** 2016-08-19

**Authors:** Kenneth Perrine, James C Gibaldi

**Affiliations:** 1 Neurological Surgery, Weill Cornell Medical College

**Keywords:** concussion, mild traumatic brain injury, somatization, post-concussion syndrome

## Abstract

This is a retrospective study of concussion patient data conducted to analyze the prevalence of somatization in patients presenting with post-concussion symptoms. Patient records from June 2010 to December 2015 were examined for concussion history, psychosocial history, neuropsychological test results, validity scores, and a symptom severity scale. Records meeting inclusion criteria from 33 males and 27 females were located. The sample had an age range of 11–78 years with a mean age of 33.40 (SD +/- 7.5 years). A clinically significant number of patients (55%) were found to be somaticizing their symptoms and a significant majority (78%) of somaticizing patients reported no loss of consciousness, retrograde amnesia, or post-traumatic amnesia but their symptom validity scales were significantly exaggerated. Caution should be exercised by clinicians to ensure that the obtained results of neuropsychological testing are reliable and valid. It is very important for the clinician to take into account the entire patient history, including psychosocial factors (such as pre-existing psychological traits or conditions) and social influences (such as stressors in family dynamics or work/school activities that may be affecting the patient's complaints).

## Introduction

Concussions are a major health problem and affect up to 3.8 million people every year in the United States. The number of reported concussions per year has doubled in the last decade [1-2]. Public awareness and media coverage of concussions has also increased dramatically in the past few years due, in large part, to the publicity surrounding the deaths of retired National Football League (NFL) football players whose brains at autopsy were found to show deposits of tau proteins, a marker of what has been termed “chronic traumatic encephalopathy” [3]. Cases of “second impact syndrome” have also been widely publicized despite research questioning the validity of this syndrome [4-5]. This publicity has resulted in a dramatic increase in the number of patients seeking consultation for concussion evaluations.

Clinics that diagnose and treat concussions and rehabilitation centers that offer comprehensive intervention services may have an unwitting tendency to overdiagnose and overtreat concussions, which usually remit spontaneously within a few weeks [6]. However, many patients present with histories of remote concussions that occurred many months and even years prior to seeking evaluation, which is far past the time frame of recovery for simple, uncomplicated concussive head injuries. These patients are usually diagnosed with “post-concussion syndrome,” which is a vague term that can include the acute and post-acute phase of several weeks or refer to lingering symptoms that do not remit in the typical course of recovery in patients [7]. Many of these patients may have somatoform or other psychiatric disorders or have secondary gain in seeking compensation. Although psychosocial factors are known to significantly impact the presence and duration of both acute and persisting concussion symptoms, few studies have examined these factors in more detail in samples of patients with lingering post-concussion symptoms. The current study was undertaken to critically analyze a consecutive cohort of patients presenting to a concussion clinic at a teaching hospital to determine psychosocial factors contributing to the resolution or maintenance of symptoms and assign the final diagnosis after a comprehensive interview and neuropsychological testing targeted for concussions.

## Materials and methods

### Sample

All consecutive patients presenting to an outpatient concussion clinic staffed by the Neurology and Neurological Surgery departments at a teaching hospital between June 2010 and December 2015 were identified by their International Classification of Diseases, 9th or 10th edition (ICD-9 or ICD-10) diagnoses. Of these 72 patients, 12 were excluded from the analysis. Exclusion from the analysis was determined by missing results for the variables of interest (n = 10) or the circumstances in which the patient was being tested (n = 2; litigation, worker’s compensation, or disability). 

### Examination of medical records

The authors reviewed medical records for concussion history taken at the time of the interview and exam, loss of consciousness (LOC), retrograde amnesia (RA), post-traumatic amnesia (PTA), psychosocial history, and scores on a neuropsychological concussion battery partially adapted from that used by the senior author in evaluating NFL and National Hockey league (NHL) professional athletes. The battery consisted of the Pittsburgh Post-Concussion Scale, Rey Auditory Verbal Learning Test, Trail Making Test, Stroop Color-Word Test, Wechsler Adult Intelligence Scale (WAIS-III) selected subtests (Digit Span, Coding and Symbol Search), Controlled Oral Word Association Test, and Grooved Pegboard task [8-11]. The Memory Complaints Inventory (MCI) was administered to assess the symptom validity of memory scores. This inventory was developed to detect exaggeration of memory complaints by comparing a patient’s results to reference groups with veridical neurologic memory disorders [12]. The senior author, a board-certified neuropsychologist, assigned diagnoses including typical sequelae of a concussion or a somatoform disorder. The subjects were determined to have a somatoform disorder through analysis of anomalies from the patient interview, extreme scores on the Pittsburgh Post-Concussion Scale, or exaggerated findings from the MCI. The study had approval from the Institutional Review Board at Weill Cornell Medical College, and all protected health information was de-identified in accordance with HIPAA’s privacy policy after the initial gathering of records. Informed consent was obtained from the participants for this study. All data was subsequently coded before analysis.

### Statistical analysis

The scores and data were coded and entered into Microsoft Excel, and all statistical analyses were carried out using SPSS for Windows (V 22.0, IBM SPSS, IBM Corporation, New York, USA). The Mann-Whitney U-test was used to compare the somaticizing and non-somaticizing groups for quantitative variables. Chi-square tests were used to compare categorical or frequency data between these two groups.

## Results

There were 60 patients with concussions (33 males and 27 females) with an age range of 11–78 years (mean = 33.4 SD+/- 7.5). Of these patients, 33 (55%) were found to be somaticizing. The mean Pittsburgh Post-Concussion Scale score on the day of the visit for somaticizing patients was 40.3 while the mean for non-somaticizing patients (16.5) was significantly lower (Mann-Whitney U = 5.00, p = 0.013). The mean scores did not differ significantly between the two groups for any of the neuropsychological tests and most were average or above. The mean time since injury for the somatoform subjects was 207.8 days (SD +/- 10.5), which was significantly greater (Mann-Whitney U *= *19.0 p = 0.004) than the group of subjects without somatoform disorders (89.45 days, SD +/- 6.5). The frequency data is presented in Table 1. A large number of patients (55%) were found to be somaticizing their symptoms. A majority (78%) of these somatoform patients reported no loss of consciousness, retrograde amnesia, or post-traumatic amnesia. A history of affective disorders was significantly more frequent (χ ^2 ^= 1.573, p = .035) in the somatoform group (81.81%) versus the non-somatoform group (18.51%). The subjects who sustained miscellaneous accidents (e.g. falls) were significantly more likely to be somaticizing than the motor vehicle- and sports-related groups (χ ^2 ^= 1.923*, *p = 0.05). Those who sustained sports-related concussions showed somatoform disorders just as frequently as those who sustained non-sports-related concussions (χ ^2 ^= 1.11, p = .603). Of the 27 females, 18 had somatoform disorders (66.67%), which was not statistically significantly different compared to the number of men (45.45%) with somatoform disorders (χ ^2 ^= 2.70, p = 0.123). The frequency of somatoform disorders did not differ significantly between self-referred (61%) versus physician referred (39%) subjects (χ ^2 ^= 0.11, p = 0.567). Education, the number of prior concussions, and age did not differ significantly between the two groups.

**Table 1:** Presentation and Outcomes *The presence of psychosocial issues is determined by analysis of the patient interviews and scores on the Minnesota Multiphasic Personality Inventory-2-Restructured Form (MMPI-2-RF). Issues noted include psychological problems such as anxiety and depression, falling into a sick role, negative expectations, bullying at school, and outside pressures.

		Number of Somaticizing Patients	Number of Non-somaticizing Patients
Sex	Male	15	18
	Female	18	9
Education	>12 years	18	15
	<12 years	15	12
Referral	Self	20	11
	MD	13	16
Psychosocial Issues*	Yes	27	5
	No	6	22
Cause	Sports-Related	4	10
	Motor Vehicle	9	10
	Other	20	7

Two cases representing examples of a somatoform disorder and a classic concussion disorder are presented below.

### Case example 1: Somatization

Subject: A 13-year-old female

History of injury: a minor blow to the head caused by a door with no LOC or amnesia. The patient was removed from full-time school and has been attending school part-time for the last six weeks.

Symptoms: Headache, photophobia, nausea, and difficulty concentrating

Post-concussion symptom scale: 74 at six weeks post-injury

Neuropsychological results: average, but of questionable validity

Psychosocial: divorced parents, father under indictment for fraud, self-reports feeling anxious

Impression: most likely fell into the sick role after being out of school for so long. The patient was advised to gradually return to school and normal routines and seek psychological support.

### Case example 2: No somatization

Subject: A 23-year-old male

History of injury: A motor vehicle accident two weeks ago with 10 min LOC, 200 min PTA, and 5 min of RA. Seeking return-to-school and return-to-play clearance.

Symptoms: Headache and fatigue

Pittsburgh Post-Concussion Scale: 15 at two weeks post-injury

Neuropsychological Results: mild deficits in processing speed but average in all other tasks

Psychosocial: stable family and social environment; self-reports no feelings of anxiety or depression

Impression: encouraged to ease back into school with breaks if necessary. Advised to gradually return to sports only when symptom-free.

## Discussion

These two cases represent the extreme ends of the spectrum, with most patients falling somewhere in between. However, they are illustrative of how non-somaticizing patients with more severe injuries can show uncomplicated and rapid recovery while patients with relatively minor injuries can endorse a large number of severe post-concussion symptoms and complaints of long-term sequelae that reportedly disrupt daily functioning. Between June 2010 and December 2015, the number of instances of somatization presented to our clinic has steadily increased from six subjects in 2011 to 17 subjects in 2014. This increase may be related, at least in part, to the overwhelming media attention that concussions have received.

Diagnosis of a somatoform disorder was based on criteria outlined by the ICD-10. These criteria include a patient’s presentation of physical symptoms in spite of negative findings by physicians and assurance that the symptoms have no physical basis. Additionally, if any physical disorder is present, such as a minor and uncomplicated head injury, it does not explain the nature and extent of the symptoms or the distress of the patient [13]. Although the time needed for the resolution of concussion symptoms is somewhat varied, there is little evidence that symptoms can be prolonged for greater than three months in a typical concussive brain injury. Deficits in cognition following a concussion are well studied and typically resolve within one week [14]. There were no significant differences in neuropsychological deficits found in patients with or without somatoform disorders, suggesting an absence of cerebral dysfunction in both groups. However, self-reported memory and post-concussion symptoms are often exaggerated in patients with a somatoform disorder who also show a much longer duration and severity of post-concussion symptoms prior to seeking evaluation. Previous studies have shown that females tend to somaticize their symptoms with more frequency than males [15] and less educated subjects tend to somaticize with higher frequency [16], but our data showed equivalent rates for gender and education.

These findings represent a critical first step in understanding the signatures and frequency of somatization in post-concussion syndrome and provide the foundational evidence to warrant further examination of these signatures through prospective studies. Ensuring a correct diagnosis is paramount in the planning of treatment for patients with post-concussion syndrome and is one of the biggest hurdles facing clinicians who treat concussions. Recent research has linked post-concussion syndrome to an increased risk of suicide and the development of depression, anxiety, and post-traumatic stress disorder (PTSD) [17-18]. It is imperative that clinicians be able to distinguish psychiatric sequelae of head injury from underlying pre-existing psychosocial issues that may manifest through the presentation of post-concussive symptoms. Further research connecting concussion with psychological disorders is needed; however, the results of this study indicate that the frequency of somatoform disorder in patients presenting with post-concussion syndrome is significant.

## Conclusions

Based on the findings of this retrospective study, it appears that somatization is fairly common in patients presenting to a concussion clinic with post-concussion syndrome. Caution should be exercised to ensure that the obtained results of clinical examinations and neuropsychological testing are reliable and valid and not reflective of somatization. A measure, such as the MCI, should be included to detect exaggeration of memory deficits, and the progression of symptoms quantified by a scale, such as the Pittsburgh Post-Concussion Scale, should be examined to determine if concussion symptoms are following a typical course or are also exaggerated. It is very important for the clinician to take into account the entire patient history, including psychosocial factors (such as pre-existing psychological traits or conditions), and social influences (such as stressors in family dynamics or work/school activities) that may be affecting the patient's complaints.

### Limitations

To mitigate any selection bias, the authors took the following steps: all patients seeking concussion evaluation during this time period were included in the cohort, and any patient who was involved in any legal proceeding was excluded in an effort to eliminate malingering for secondary gain as a possible confound. Retrospective analyses are vulnerable to biases and a prospective study is currently being undertaken to resolve this problem. The diagnosis of a somatoform disorder or typical sequela of concussion was based partially on some of the tests used in the analyses, which may have presented a confound. Small cohorts have a tendency to lack statistical power when studying rare syndromes and diseases. However, due to the high number of somatoform patients presenting with concussions, the results obtained in this study may be clinically significant and warrant further research. 

### Disclosure

Dr. Perrine is a consultant to the New York Jets and the New York Islanders.
